# Lateralized tactile stimulation during NREM sleep globally increases both slow and fast frequency activities

**DOI:** 10.1111/psyp.14191

**Published:** 2022-09-25

**Authors:** Péter Simor, Tamás Bogdány, Rebeca Sifuentes‐Ortega, Antonin Rovai, Philippe Peigneux

**Affiliations:** ^1^ Institute of Psychology ELTE Eötvös Loránd University Budapest Hungary; ^2^ UR2NF, Neuropsychology and Functional Neuroimaging Research Unit at CRCN—Center for Research in Cognition and Neurosciences Brussels Belgium; ^3^ UNI—ULB Neurosciences Institute Université Libre de Bruxelles (ULB) Brussels Belgium; ^4^ Doctoral School of Psychology ELTE Eötvös Loránd University Budapest Hungary; ^5^ Laboratoire de Cartographie fonctionnelle du Cerveau (LCFC), ULB Neuroscience Institute (UNI), CUB‐Hôpital Erasme Université libre de Bruxelles (ULB) Brussels Belgium; ^6^ Department of Functional Neuroimaging, Service of Nuclear Medicine, CUB‐Hôpital Erasme Université libre de Bruxelles (ULB) Brussels Belgium

**Keywords:** brain stimulation, EEG, laterality, sleep, slow waves

## Abstract

Slow frequency activity during non‐rapid eye movement (NREM) sleep emerges from synchronized activity of widely distributed thalamo‐cortical and cortico‐cortical networks, reflecting homeostatic and restorative properties of sleep. Slow frequency activity exhibits a reactive nature, and can be increased by acoustic stimulation. Although non‐invasive brain stimulation is a promising technique in basic and clinical sleep research, sensory stimulation studies focusing on modalities other than the acoustic are scarce. We explored here the potential of lateralized vibro‐tactile stimulation (VTS) of the finger to locally modify electroencephalographic activity during nocturnal NREM sleep. Eight seconds‐long sequences of vibro‐tactile pulses were delivered at a rate of 1 Hz either to the left or to the right index finger, in addition to a sham condition, in fourteen healthy participants. VTS markedly increased slow frequency activity that peaked between 1–4 Hz but extended to higher (~13 Hz) frequencies, with fronto‐central dominance. Enhanced slow frequency activity was accompanied by increased (14–22 Hz) fast frequency power peaking over central and posterior locations. VTS increased the amplitude of slow waves, especially during the first 3–4 s of stimulation. Noticeably, we did not observe local‐hemispheric effects, that is, VTS resulted in a global cortical response regardless of stimulation laterality. VTS moderately increased slow and fast frequency activities in resting wakefulness, to a much lower extent compared to NREM sleep. The concomitant increase in slow and fast frequency activities in response to VTS indicates an instant homeostatic response coupled with wake‐like, high‐frequency activity potentially reflecting transient periods of increased environmental processing.

## Abstract 2

Non‐invasive brain stimulation is a promising technique to enhance slow wave sleep. We applied lateralized vibro‐tactile stimulation to locally increase slow waves during NREM sleep. We evidenced a global effect of increased slow and faster frequency activity, with no signs of a lateralized effect. The concomitant increase in slow and fast frequency activities indicates an instant, NREM‐specific homeostatic response coupled with wake‐like activity potentially reflecting transient periods of increased environmental processing.

## 1 INTRODUCTION

Non‐rapid eye movement (NREM) sleep is the most dominant sleep state in human adults, covering about 70% of night‐time sleep. NREM sleep is shaped by homeostatic, circadian, and ultradian regulation leading to the cyclic alternation of gradually deepening and lightening periods of sleep (Carskadon & Dement, 2005). NREM sleep features a variety of interrelated, short‐lasting cortical activities such as vertex sharp waves, K‐complexes, sleep spindles, and slow waves. These graphoelements as captured by electroencephalography (EEG) seem to play prominent roles in the fine balance between sensory disconnection and environmental alertness (Halasz & Bodizs, 2012), and reflect the restorative properties of NREM sleep (Fultz et al., 2019; Rasch & Born, 2013; Tononi & Cirelli, 2006). More specifically, low frequency EEG oscillations including delta (0.5–4 Hz) and theta (4–8 Hz) frequencies reflecting the synchronized activity of widely distributed thalamo‐cortical and cortico‐cortical networks, and large (>75 mV) amplitude neocortical slow oscillations (SO) around 1 Hz are considered to be critical components contributing daytime cognitive processes such as executive functions, learning, and memory consolidation (Bellesi et al., 2014; Dang‐Vu et al., 2006; Wilckens et al., 2018).

Although low frequency activity indicates a quiescent state under environmental disconnection, and increased thresholds for arousal and awakening, slow oscillatory activity exhibits a reactive nature (Halász et al., 2014), and can be externally elicited during sleep by cost‐effective, non‐invasive techniques such as acoustic stimulation (Bellesi et al., 2014). Accordingly, a growing number of studies evidenced the potential of boosting slow wave activity by repetitive acoustic stimulation during NREM sleep (Garcia‐Molina et al., 2018; Ngo et al., 2013, 2015; Santostasi et al., 2016). Enhancing slow wave activity during NREM sleep is considered to be a promising approach to facilitate sleep‐dependent memory consolidation (Ngo et al., 2013; Ong et al., 2016), as well as to ameliorate cognitive decline in healthy and pathological aging (Diep et al., 2020; Papalambros, Malkani, et al., 2017; Papalambros, Santostasi, et al., 2017); however, its efficacy in real‐life and clinical settings is far from being established (Henin et al., 2019; Wunderlin et al., 2020).

Although information processing during sleep is not limited to the auditory pathway, studies focusing on modalities other than the acoustic are scarce. Previous studies indicate that rhythmic vestibular stimulation may also increase slow frequency activity and deepen sleep during a daytime nap (Bayer et al., 2011). On the other hand, repetitive visual stimulation was not effective to enhance slow wave activity in contrast to acoustic stimulation of similar rhythmicity (Danilenko et al., 2020). The sleeping brain is particularly sensitive to information arising from the body (Wei & Van Someren, 2020), therefore, slow waves might be effectively elicited through the somatosensory pathway. Two previous studies that applied repeating electrical (Veldman et al., 2021) and mechanical (Pereira et al., 2017) stimulation on the skin (of the index fingers) within the frames of targeted memory reactivation paradigms provided indirect data on the effect of tactile stimulation on sleep EEG. Their findings however are rather inconsistent, as Pereira et al. (2017) reported an increase in SO and decrease in spindles in response to stimulation, whereas Veldman et al. (2021) observed increased sigma (i.e., in the frequency range of spindles) and beta power, and reduced delta power. As the stimulation of the fingers in these studies was related to specific finger‐tapping sequences performed before falling asleep (that is, to pre‐sleep learning experience), it is not possible to disentangle whether these changes in frequency‐specific activities merely reflect the cortical response to rhythmic stimulation, or neural processes underlying synaptic plasticity and memory consolidation related to the reactivation of previous learning experience, or both.

Our aim here was to examine the possibility of entraining slow oscillatory activity during NREM sleep by repetitive vibro‐tactile stimulation (VTS) that was not related to previous (pre‐sleep) learning. Slow oscillatory activity during sleep is expressed in a region‐specific manner (Cajochen et al., 1999; Ferrara et al., 2002), and shows relative increases over regions that were exposed to higher information‐processing demands (Hung et al., 2013). Moreover, interhemispheric asymmetries were also observed in the expression of slow wave sleep in birds (Rattenborg et al., 2000), mammals (Reicher et al., 2021) and to some extent in humans (Cajochen et al., 2008; Tamaki et al., 2016). In light of these local aspects of slow wave sleep, we hypothesized that lateralized VTS would lead to asymmetric appearance of reactive slow waves, that is, relatively increased slow frequency activity in the contralateral hemisphere.

## 2 METHODS

### 2.1 Participants

Participants were recruited through online announcements at the Universite Libre de Bruxelles (ULB, Belgium). And 17 healthy young adults spent a full night of sleep in the sleep laboratory at the ULB Neuropsychology and Functional Neuroimaging Research Group (UR2NF). They were all right‐handed, without any prior or current psychiatric, neurological, chronic somatic disorders, signs of depressive symptoms (Beck's Depression Scale ≥12, Beck et al., 1988), or sleep complaints (Pittsburgh Sleep Quality Index ≥4, Buysse et al., 1989). Regular sleep schedules for at least 1 week before the experiment were verified using daily sleep logs and actimeters. Before the night spent in the laboratory, participants were asked to go to sleep one hour later and wake up one hour earlier than their normal sleep schedule. This partial sleep deprivation was intended to stabilize their sleep during the laboratory night when they received stimulation. Data of 3 participants were excluded due to technical problems (*n* = 2), or increased sensitivity to the stimulation procedure (*n* = 1) (i.e., reduced time spent in NREM sleep due to arousals and awakenings in response to stimulation). Consequently, night‐time sleep EEG data were analyzed for 14 participants (9 females, mean age = 24.38 yrs, *SD* = 3.67). An additional wake control condition group (*N* = 15, 14 females, age = 24.93 yrs, *SD* = 3.61) received the same tactile stimulation procedure as the sleep group, but during quiet resting wakefulness (in supine position with eyes closed). Participants received monetary compensation for taking part in the experiment. The study procedure was carried out in accordance with the Declaration of Helsinki, and approved by The Ethical Committee of the Universite Libre de Bruxelles.

### 2.2 Procedure

Participants arrived to the lab between 20.00–21.00 and were fitted with Ag/AgCl electrodes at 19 scalp locations following the 10–20 standard rules for the placement of electrodes (Keil et al., 2014). Scalp electrodes were referenced to the average of the mastoids. Bipolarly referenced electrooculography (EOG) and submental electromyography (EMG) were also used. Impedances were kept below 10 kΩ. Signals were collected, amplified, and digitized at 512 Hz sampling rate with 24‐bit resolution with the 32 channel SD LTM Express, Morpheus (Micromed) EEG system and the SystemPlus Evolution software (Micromed, Mogliano Veneto, Italy). High‐pass and low‐pass filters were set at 0.3 and 48 Hz for visualization, respectively, for the EEG and EOG channels, and at 10 and 100 Hz for the EMG channels. Vibro‐tactile stimulators were fixed to the left and right index fingers. Before starting night‐time recordings, we verified that participants could feel the stimulations on their fingers. The trigger stimulation device was designed and built in‐house using an Arduino board and round 3 V vibrators (diameter: 10 mm, thickness: 3 mm), each vibrating at approximately 200 Hz. The Arduino was controlled by a MATLAB program connected through serial communication at 115,200 baud rate. Lights‐off was scheduled between 22.30 and 00.30 adapted to the participants' usual sleep habits. VTS (detailed below) was performed during the night. Awakening was scheduled between 6.30 and 8.30, adapted to the participant's usual waking hours. After awakening, we tested whether tactile stimulations were still perceived by the participants. They were also queried whether they felt/remembered any repetitive stimulations during the night. None of the participants included in our analyses reported to feel the stimulations during the night, except at the moments of awakenings, when the stimulations were directly stopped. Participants in the wake control condition underwent the same procedure, but recordings were scheduled between 11.00 and 14.00. Participants spent 70–80 min in resting supine position while they received the stimulations.

### 2.3 Vibro‐tactile stimulation

During the night, researchers constantly monitored sleep EEG. They started the stimulation procedure whenever the participant spent at least 10 min of undisturbed sleep in Stage 2 or SWS (slow wave sleep) during the night. In the first sleep cycle however, the first series of stimulations could only be started upon the appearance of SWS to avoid abrupt awakenings in the beginning of the night (compromising the quality of sleep) and allow participants to enter already into stabile, deep sleep. Each VTS trial lasted 8 s, consisting of eight 100 ms long pulses sent at 1 Hz rate (i.e., inter‐stimulus interval was set to 900 ms) following previous acoustic stimulation procedures (Simor et al., 2018). The 8‐s long stimulation trial was always followed by a silent, non‐stimulated period lasting 10–15 s until the start of the next stimulation trial. The duration of these silent periods was not fixed in order to minimize the anticipation of the upcoming stimulation. Each trial was randomly sent to the left hand, to the right hand, or to none (sham condition) of the hands. In case of the sham condition, no stimulation was sent, but triggers marking the time points of (virtual) stimulations were recorded in the time series the same way as in the right and left conditions. The experimenter stopped the stimulation in case of awakenings or longer (>5 s) arousals, and restarted the stimulation only when the participant returned into stage 2 or SWS. VTS was delivered in a similar manner in the wake control condition, including left, right, and sham conditions.

### 2.4 Data analysis

#### 2.4.1 Preprocessing

Data analysis was performed in MATLAB (version 8.3.05.32, R2014a, The MathWorks, Inc., Natick, MA) using the FieldTrip toolbox (Oostenveld et al., 2011). In order to verify whether the trials were accurately sent in NREM sleep, a trained sleep specialist (blind to the timing of stimulations) scored the night‐time sleep recordings following standardized criteria (Berry et al., 2012); only trials that corresponded to periods scored as Stage 2 or SWS were considered in our analyses. Stimulation trials (right, left, and sham) sent during Stage 2 and SWS were selected for further analyses. Selected trials consisted of 12 s long time series of EEG data including a 4 s long baseline (silent) period before the 8 s long stimulation trial. Sleep EEG recordings were pre‐processed and band‐pass filtered between 0.3 and 70 Hz (with Butterworth, zero phase forward and reverse digital filter). All selected trials were visually inspected; trials with technical or movement‐related artifacts were discarded. After the removal of trials with artifacts, our sleep database consisted of an average of 32.64 (range: 11–56), 33.19 (range: 15–57), and 29.28 (range: 4–52) trials, for the left, right, and sham conditions, respectively. Our wake control database consisted of an average of 51.86 (range: 44–60), 38.26 (range: 15–57), and 34.73 (range: 24–49) trials, for the left, right, and sham conditions, respectively. Independent component analysis (ICA) of the sleep and resting wake segments (concatenated over left‐sided, right‐sided, and sham conditions) was performed to identify cardiac, eye‐movement, and other muscular artifacts using Fieldtrip routines (Oostenveld et al., 2011). Independent components (in sleep mostly one, maximum two, in resting wakefulness mostly two‐three, maximum four) representing components linked to eye movements and muscular artifacts were detected semi‐automatically and were identified by inspecting the waveforms, as well as their topographical distribution (Campos Viola et al., 2009).

#### 2.4.2 The influence of VTS on EEG power

In order to examine whether VTS elicited changes in EEG power (total power), we performed time‐frequency analyses on right, left, and sham trials using Fast Fourier Transformation (FFT) of overlapping, Hanning‐tapered, 2 s long periods (0.5 Hz frequency resolution between 1 and 30 Hz) sliding in 50‐ms steps throughout the selected 12 s trials (yielding 97.5% overlap across the consecutive 50‐ms long time windows). The first 4 s were used as a baseline before the onset of stimulations, and changes in EEG power were defined relative to this baseline. The trials of time‐frequency changes were averaged (within condition and within participants) for subsequent statistical comparisons (within‐participant design). Changes in frequency‐specific power after the onset of stimulations compared to similar frequency‐specific power changes observed in the sham conditions were contrasted by cluster‐based permutation statistics across all participants. Cluster‐based permutation statistics is suitable for the analyses of EEG signals as it does not require assumptions of data distribution and is able to address the issue of multiple comparisons (Maris & Oostenveld, 2007).

In brief, one‐tailed paired samples *t* tests were performed for all pairs of data points in time and frequency bins. We ran one‐tailed *t* tests to compare the two trial types because we expected a relative increase in slow frequency power during the stimulation compared to the sham condition. More specifically, we expected that in the stimulation conditions slow frequency power will increase compared to the pre‐stimulation periods, whereas we did not expect such increases in the sham conditions. To reduce the multidimensionality of our data and to focus our analyses first on the frequency‐specific effects of stimulation, we averaged our values over all the EEG channels and focused first on the time and frequency dimensions (analyses taking into account the three dimensions of space, frequency, and time are reported among the Supplementary materials). Clusters were defined if at least two adjacent time points or neighboring frequency bins showed significant differences at the alpha level below .05. These observed clusters were selected to compute the observed cluster statistic defined by the sum of all the *t*‐values that formed a given time‐frequency cluster. The same process was repeated 1000 times by randomly shuffling stimulated and sham conditions (using MonteCarlo simulations). From these simulations the largest clusters were extracted in order to create a distribution of the maximal clusters produced by chance. Finally, the observed cluster statistics were tested (with an alpha value of .05) against the probability distribution of the largest simulated clusters. Although we averaged our values over the EEG channels to focus on time‐frequency clusters, we explored the topographical aspects of specific clusters that emerged in the time and frequency dimensions differentiating the stimulated from the sham trials. We extracted and compared the antero‐posterior gradients (Knyazev, 2010) to examine further if the spatial dimension of the obtained clusters were different. First, we extracted the contrast matrices of time frequency power (channels × time) between the stimulated and sham conditions, and averaged along the observed cluster of frequencies, separately for each cluster. Next, we averaged the channel data along five latitudinal dimensions: (1) frontopolar (Fp1, Fp2); (2) frontal (F7, F8, Fz, F3, F4); (3) central (T3, C3, Cz, C4, T4); (4) parietal (T5, P3, Pz, P4, T6); and (5) occipital (O1, O2). Finally, differences between all but the last variable and the next one were calculated and averaged, yielding to a final vector of antero‐posterior (slow and fast frequency) gradients along the time axis (Knyazev, 2010), where positive values indicate higher power over anterior regions compared to more posterior ones. The antero‐posterior gradients of the observed clusters were visualized and compared by non‐parametric permutation tests.

Next, we aimed to explore if the side of the stimulation had a lateralized effect, that is, if the side of the stimulation had a differential effect over the contralateral versus the ipsilateral hemisphere. In order to examine if stimulation produced a relatively larger change in EEG power over the corresponding (contralateral) hemisphere, we compared stimulation‐induced changes (compared to baseline) in time‐frequency power between the targeted and untargeted hemispheres. In order to focus on the side‐specific effects of the stimulation as well as to reduce the dimensionality of the data, we averaged the change in power over the targeted and untargeted hemispheres (analyses taking into account the three dimensions of space, frequency, and time are reported among the Supplementary materials). Hence, if we stimulated the right finger, the so‐called targeted hemisphere corresponded to signals measured at left‐sided channels (Fp1, F3, F7, C3, T3, T5, P3, O1), and if we stimulated the left finger, the targeted hemisphere corresponded to signals measured at right‐sided channels (Fp2, F4, C4, T4, T6, P4, O2), whereas activity in midline channels were not considered in these analyses. Changes in time‐frequency power across the targeted versus the untargeted hemispheres were evaluated by cluster‐based permutation statistics, using 1000 random partitions to create the random distribution of clusters for statistical reference. We assumed that stimulation would elicit a relatively larger increase in slow frequency power over the targeted than the untargeted hemisphere. As it is theoretically feasible that lateralized stimulation does not exert a similar effect in left and right sided stimulations, we performed two cluster‐based permutation tests contrasting power changes over the targeted and the untargeted sides in case of left and right sided stimulation, separately.

#### 2.4.3 Changes in the amplitude of slow waves in response to VTS

We also examined if VTS increased the amplitude of evoked slow waves time‐locked to the onset of trials. Therefore, we averaged the 12 s long segments to extract the event related potentials (ERPs) time locked to the stimulation (with 4 s long pre‐stimuli baseline periods and 8 s of stimulation), separately for the left, the right and sham conditions in each participant. To contrast ERPs between stimulated and sham periods, as well as the ERPs between targeted and untargeted hemispheres, we computed the magnitude (absolute values) of evoked voltage fluctuations over the 12 s long segments. We computed the absolute values of evoked potentials to discard negative deflections and to compare the amplitude of evoked waveforms regardless of up‐or downward deflections of oscillatory activity. The absolute values of ERPs were corrected for the baseline, and the baseline‐corrected magnitudes of evoked potentials were compared across the specific conditions by cluster‐based permutation statistics in our sample of participants using 1000 random partitions (shuffling the labels of the conditions). In this case, we averaged our values over the time axis, but not over electrode sites.

In addition, we aimed to examine if the effect of stimulation varied as a function of the order of stimulation trials. For instance, if the first set of stimulations had stronger influence on EEG power changes compared to subsequent stimulation trials. Therefore, we averaged EEG power changes in response to stimulation over the 8 s stimulated periods and within the clusters of frequencies that differentiated the stimulation and sham conditions, and extracted these averaged relative power values for each trial. To examine the influence of the order of stimulation trials during the stimulation procedure we performed two separate linear mixed‐model analyses including EEG power changes (relative to the baseline) of all trials as the outcome variables. In the first model, we evaluated the influence of time on the effect of Stimulation; hence, predictors were the trial number, termed as Order (a numeric value from 1 to the length of the number of trials), Condition (Stimulation vs. Sham), and Band (including two clusters: slow and fast frequency activities). In the second model, we examined if time had an influence on the effect of stimulation over the targeted versus the untargeted hemisphere, therefore, we included only stimulation trials in our dataset, and evaluated the fixed effects of the predictors: Order, Site (Targeted vs. Untargeted), and Band (including two clusters: slow and fast frequency activities). Models included random intercept and random slope for each predictor, among which the latter were removed if the model did not converge. Mixed‐model analyses were performed in Jasp (version 0.14.1 (Team (2020), 2020)).

## 3 RESULTS

### 3.1 Sleep architecture

Sleep architecture indices are presented in Table 1. Overall, participants exhibited high sleep efficiency, short sleep latency, and the time spent in NREM stages and in REM sleep did not deviate from standard norms for similar age groups (Hertenstein et al., 2018), indicating that the stimulation procedure did not compromise sleep quality in this sample of participants.

**TABLE 1:** Sleep architecture of the night time recordings in our sample of participants

	Mean (SD)	Range
Sleep duration (min)	448.0 (64.7)	370.7–560.0
Sleep efficiency (%)	92.1 (5.0)	85.6–96.5
Sleep latency (min)	4.3 (5.2)	2.1–16.3
Wake after sleep onset (min)	32.3 (20.2)	2.0–67.3
Stage 1 (%)	5.5 (2.9)	2.0–11.8
Stage 2 (%)	55.2 (7.7)	45.1–67. 3
SWS (%)	16.9 (4.1)	10.9–24.8
REM (%)	22.4 (5.0)	15.8–29.7
REM latency (min)	107.8 (33.7)	65.1–240.3

### 3.2 EEG power changes in response to vibro‐tactile stimulation

**FIGURE 1:**
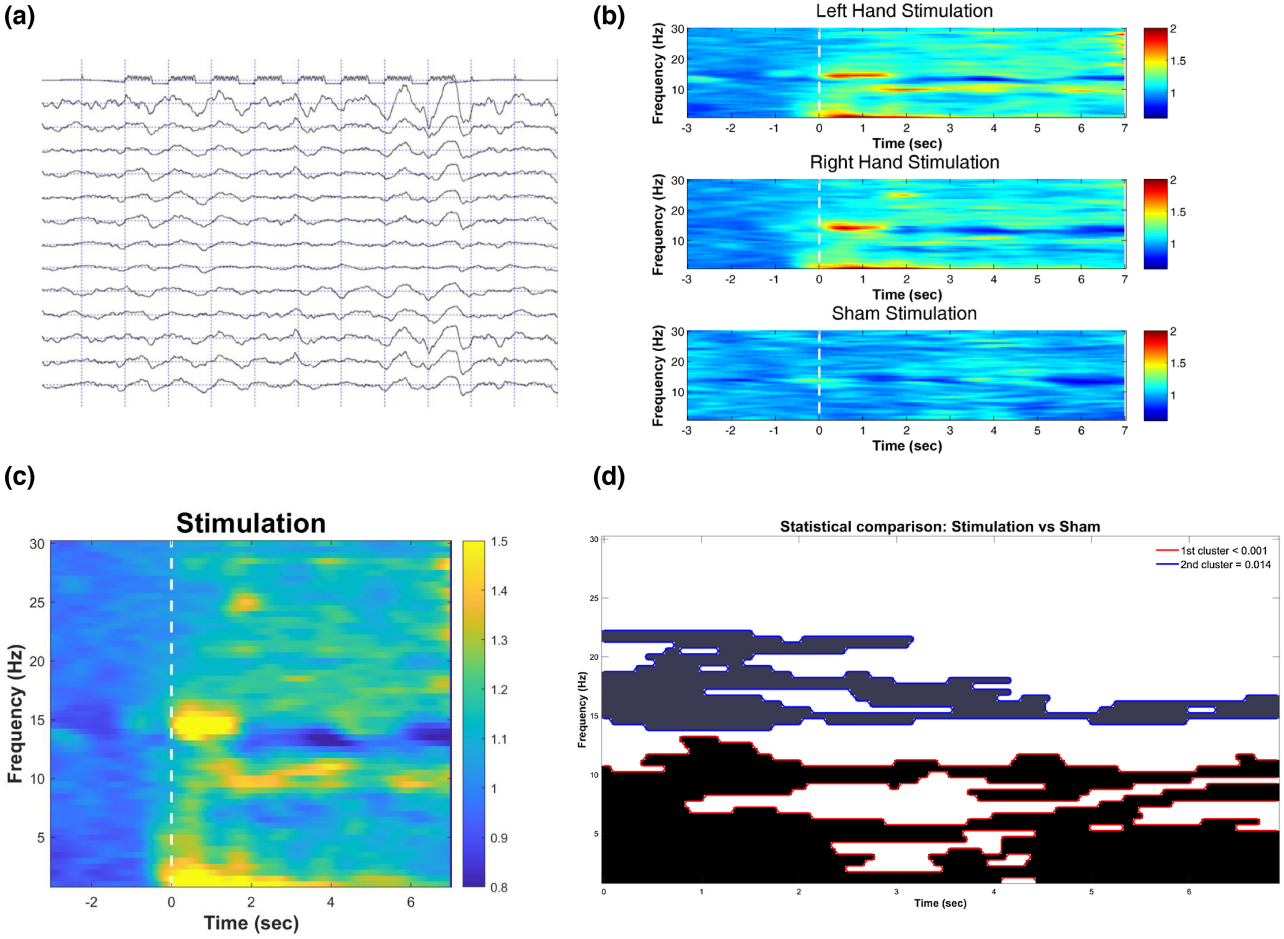
The effect of vibro‐tactile stimulation on EEG power changes during NREM sleep. (a) Raw recording showing the onset of vibro‐tactile stimulation, indicated by 8 pulses of repetitive vibrations delivered with 1 Hz frequency. The first channel shows the timing of the stimulations during which an increase in slow waves is observable on the EEG channels (we show a reduced number of EEG channels for visualization purposes). (b) Time‐frequency power changes during stimulation to the left and the right hand, and during sham conditions. The white dashed lines indicate the onset of the trials. An increase in slow and fast frequency power is apparent in both left‐ and right‐sided stimulation compared to the sham trials. Color codes between 0.8 and 1.8 represent the power ratio between stimulation and sham conditions. (c) Time‐frequency power changes during stimulation (mean of left and right sided trials). The white dashed line indicates the onset of the trials. (d) Clusters in the time and frequency dimensions differentiating stimulation versus sham conditions with respect to EEG power changes after the onset of 8 s long stimulations.

First, we verified whether the side of the stimulation (left or right) had different influence on EEG power changes. Therefore, we contrasted the relative change in power (compared to the baseline) in left‐ versus right‐sided stimulation trials. In order to detect any differences between the two conditions, in this case, we did not reduce the multidimensionality of our data, and performed our comparisons in the dimensions time, frequency, and topography. As we had no previous assumptions about the differences between stimulations on the left and the right sides, *t* tests forming the clusters were set to two tailed, paired *t* tests. We did not detect clusters that were significant (largest cluster *T*_sum_ = −561.2; cluster level *p* value > .4, CI_range_ = 0.03), thus, no differences emerged in EEG power changes as a function of the side of the stimulation (for a visualization see Figure 1b).

To examine if the stimulation had an effect on EEG power we aggregated the left‐ and right‐sided trials and contrasted them with the sham trials. Two main clusters emerged that differentiated the stimulation and the sham trials. The first cluster (*T*_sum_ = 6215.1; cluster level *p* value < .001, CI_range_ = 0.002, Cohen's *d*_range_: 0.47–1.79) comprised slower frequencies between 1 and 13 Hz, and spanned over the whole time range, showing peaks at slower components between 1–4 Hz, especially in the first three seconds of the 8 s long stimulation sequences (see Figure 1c,d). The second cluster (*T*_sum_ = 2741.8; cluster level *p* value = .014, CI_range_ = 0.007, Cohen's *d*_range:_ 0.47–1.38) included faster frequencies between 14 and 22 Hz showing a short‐lasting (~1 s) peak around 15 Hz, but then spanning over the 8 s stimulation period with an apparently even distribution (Figure 1c,d).

For the wake condition we performed the same analyses as above to examine if the observed changes in power are different across sleep and wake. Similarly, to NREM sleep, no significant differences emerged across left‐ and right‐sided stimulations in the wake condition (largest cluster *T*_sum_ = 92.09; cluster level *p* value > .5, CI_range_ = 0.009). Statistical comparison of the stimulated (left and right) and sham conditions yielded two clusters (*T*_sum_ = 1040.3; cluster level *p* value = .025, CI_range_ = 0.01, Cohen's *d*_range:_ 0.45–1.97; *T*_sum_ = 998.5; cluster level *p* value = .026, CI_range_ = 0.01, Cohen's *d*_range:_ 0.4–1.24) that survived the correction for multiple comparisons. The first cluster comprised slow frequencies between 1 and 6 Hz and the second between 16 Hz and 23.5 Hz, and spanned from the onset of stimulation to the 2.5 and 4.5 s, respectively (See Figure S1a–c). In order to directly compare if VTS had differential influence on time frequency power changes during sleep, we contrasted and statistically compared power changes in response to stimulation across the sleep and the wake group. That is, baseline corrected time frequency power averaged across left‐ and right‐sided stimulation were compared between the two vigilance states following the same statistical procedure as described above (i.e., time × frequency matrices were contrasted after averaging in the spatial dimension). VTS during sleep was significantly different than VTS in wakefulness with regard to induced changes in time frequency power. Statistical comparison of the sleep and the wake group yielded three positive clusters that survived the correction for multiple comparisons. Clusters (indicating more pronounced changes in power in the sleep vs the wake group) comprised frequencies between 1 and 12 Hz (*T*_sum_ = 3897; cluster level *p* value < .001, CI_range_ = 0.002, Cohen's *d*_*r*ange:_ 0.76–1.74), between 13.5 and 22 Hz (*T*_sum_ = 4954.2; cluster level *p* value < .001, CI_range_ = 0.002, Cohen's *d*_range:_ 0.76–1.91), and between 25.5 and 30 Hz (*T*_sum_ = 1000.6; cluster level *p* value = .027, CI_range_ = 0.01, Cohen's *d*_range:_ 0.76–1.56). These findings clearly indicate that VTS had a more pronounced effect on cortical activity during NREM sleep as assessed by changes in time frequency power (See Figure S2).

### 3.3 The increase in slow and fast frequencies shows different topographical distributions

**FIGURE 2:**
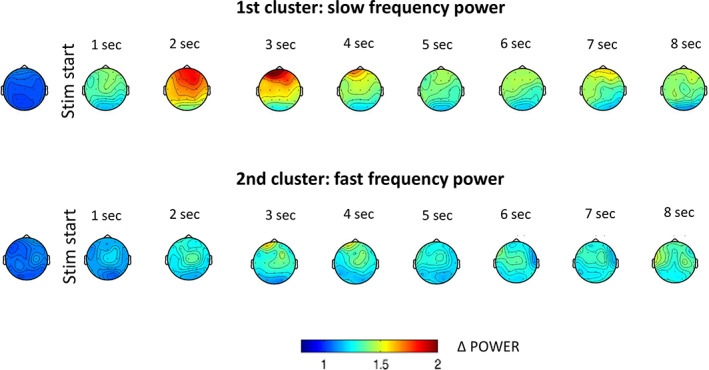
Topographical aspects of the increase in slow and fast frequency power during vibro‐tactile stimulation in NREM sleep. The relative increase in slow (1–13 Hz) frequency (top row) and fast (14–22 Hz) frequency (bottom row) EEG power during stimulation trials in contrast to sham conditions. Headplots show relative power values one second before (first headplot) and during the eight seconds of the stimulation trials. Color codes represent the power ratio between stimulation and sham conditions.

We inspected the topographical aspects of the two clusters that significantly differentiated the stimulation and the sham conditions. The first cluster reflecting the stimulation‐driven increase in low frequency EEG power showed a fronto‐central distribution, peaking mainly over frontal electrode sites especially in the first 3–4 s of stimulation. On the other hand, the second cluster reflecting the increase in fast frequency activity due to stimulation was first observed over central sites and then was extended all over the scalp (Figure 2).

**FIGURE 3:**
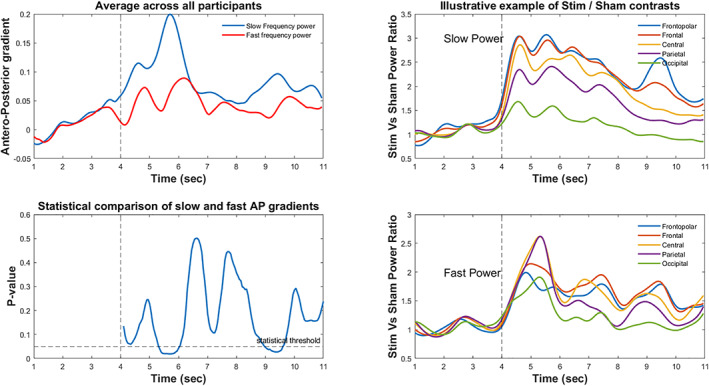
Antero‐posterior gradients of slow and fast frequency power changes in response to stimulation in NREM sleep. Antero‐posterior gradients show higher values in the observed slow frequency cluster than the fast frequency cluster (left column). Positive values on the left upper graph indicate relatively increased power changes in response to stimulation over anterior as compared to more posterior electrode sites. Left lower graph shows the respective *p*‐values of statistical comparisons between anterior gradients of slow and fast frequency clusters along the time axis. On the right, an illustrative example of one participant indicates the relative power changes (stimulation/sham) averaged across the latitudinal dimensions for the slow (right upper figure), and the fast (right lower figure) frequency clusters, respectively.

To further explore the topographical differences between the observed slow and fast frequency clusters, we compared the antero‐posterior gradients of these slow and fast frequency contrasts. As shown in Figure 3, the relative increase in slow frequency power in response to the stimulation showed a more pronounced antero‐posterior gradient than the relative increase in fast frequencies. Significant differences (*p* < .05; CI_range_ ~ 0.02, Cohen's *d*_range:_ 0.5–0.62) were observed in the beginning (2nd second) and end (6th second) after the stimulation (see Figure 3). Interestingly, peaks of the antero‐posterior gradient showed a fluctuation appearing with a (0.5–1 s) delay after the pulses of VTS (see Figure 3 for the average of antero‐posterior gradients and an illustrative example of the data of one participant along the extracted latitudinal dimensions).

### 3.4 No signs of relative EEG power increases in the targeted hemisphere

**FIGURE 4:**
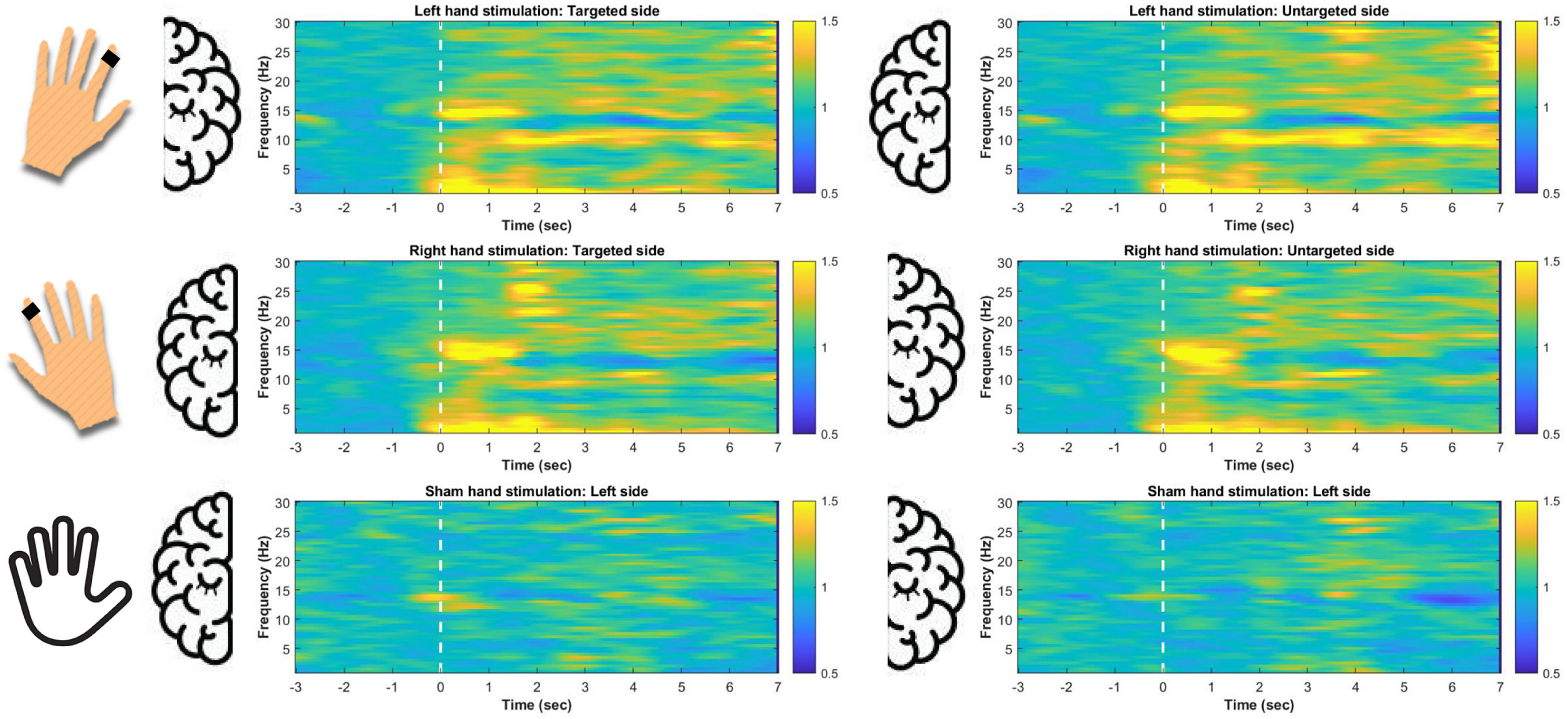
Lateralized stimulation on the targeted and untargeted hemispheres in NREM sleep. EEG power changes in response to stimulation delivered to the left and the right hand, compared to sham conditions. White dashed vertical lines indicate the onset of stimulations. Heatplots show the averaged EEG power changes over the targeted (contralateral) and untargeted (ipsilateral) hemispheres. EEG power changes in sham conditions averaged over the left and the right hemispheres are also visualized. While vibro‐tactile stimulation had a marked effect on EEG power, no significant differences were observed across the targeted and untargeted hemispheres.

We compared the relative increase in EEG power in response to stimulation over the targeted (contralateral) and untargeted (ipsilateral) hemispheres separately for right‐and left sided stimulation. No clusters that significantly differentiated EEG power changes over the targeted and untargeted hemispheres emerged (right hand stimulation: no clusters were identified; left hand stimulation: largest cluster *T*_sum_ = 414.67; cluster level *p* value = .08, CI_range_ = 0.01). The largest cluster showing a trend in case of stimulation of right hand comprised frequencies between 18 and 21.5 Hz over the time range between 4 and 7 s. Beyond this trend our analyses provide no robust evidence for a lateralized effect of VTS as a function of the targeted side (see Figure 4 and Supplementary materials). In order to address more directly whether and interaction emerged between the side of the stimulation and the relative increase in power across the two hemispheres, we performed a complementary cluster‐based permutation test. In this analysis, induced changes in time frequency power in response to the stimulation in the targeted and untargeted hemispheres were divided by the induced changes in time frequency power in the sham condition within the corresponding hemispheres. That is, power changes in the targeted and untargeted hemispheres were corrected by the power changes in the corresponding hemispheres during the sham conditions. Such analysis is considered to be more sensitive to formally test for an interaction between the Condition (Stimulation vs. Sham) and the stimulated side (Targeted vs. Untargeted)(Oostenveld et al., 2011). Left‐ and right‐sided data were averaged for both the targeted and untargeted hemispheres which were then contrasted with cluster‐based permutation test. No significant clusters emerged that differentiated change in power across the targeted and untargeted hemispheres (largest cluster *T*_sum_ = 70.39; cluster level *p* value = .8, CI_range_ = 0.02). No significant differences emerged across the targeted and the untargeted hemispheres in the control resting wakefulness condition either (largest cluster *T*_sum_ = 80.76; cluster level *p* value > .5, CI_range_ = 0.02).

### 3.5 Amplitude fluctuations time‐locked to vibro‐tactile stimulation

**FIGURE 5:**
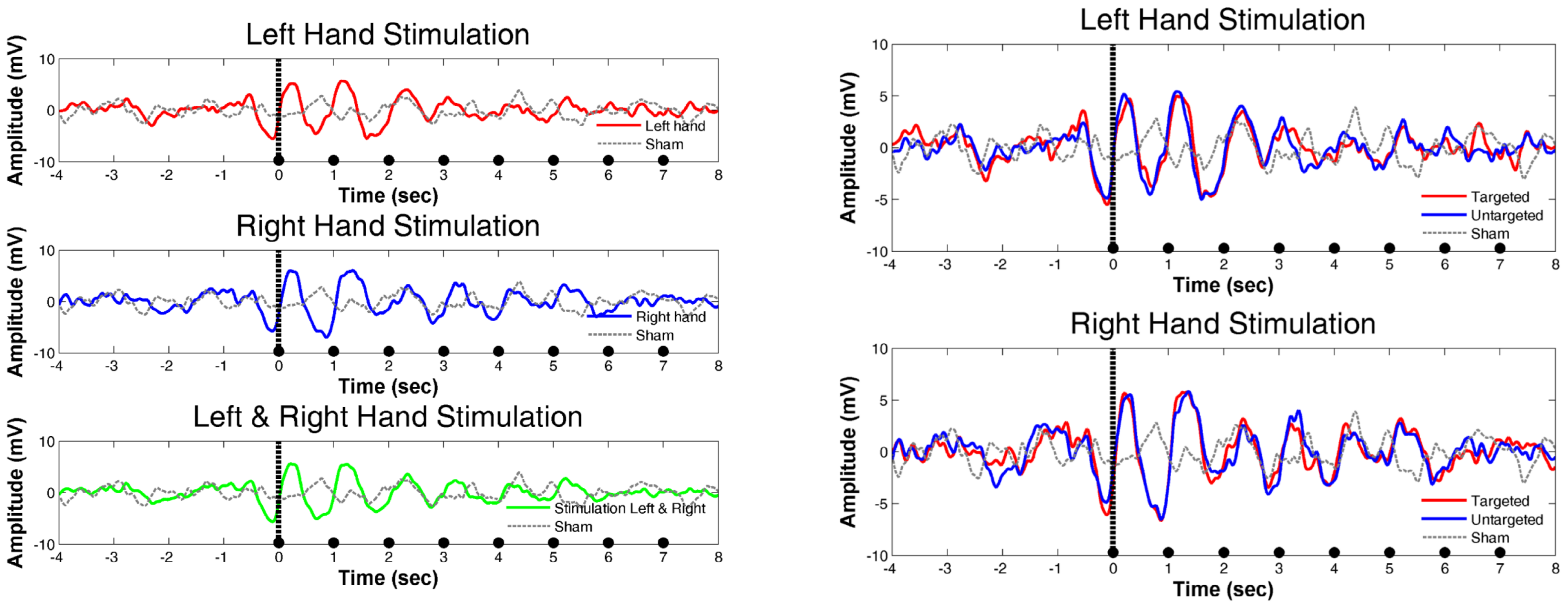
Amplitude fluctuations time‐locked to lateralized vibro‐tactile stimulation during NREM sleep. Event‐related potentials time‐locked to left‐ and right‐sided vibro‐tactile stimulations contrasted to sham conditions. On the left, ERPs are averaged across all electrode derivations, on the right ERPs are averaged for the targeted (contralateral) and untargeted (ipsilateral) hemispheres, compared to the average of the corresponding electrodes in the sham conditions. The black dashed vertical lines mark the onset of the stimulation sequences, black points on the *x*‐axis indicate the specific vibro‐tactile pulse onsets.

**FIGURE 6:**
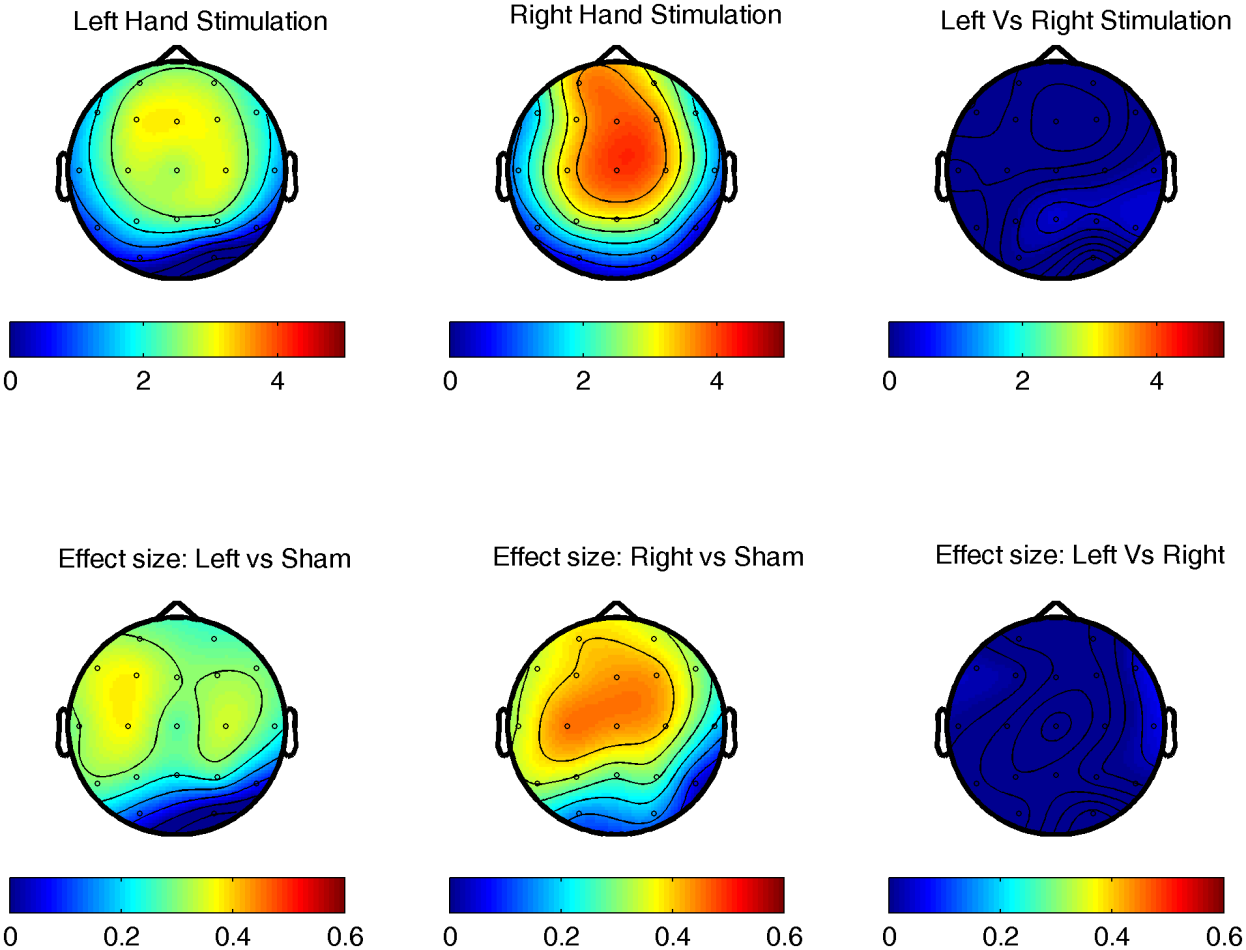
The magnitude of event‐related potentials contrasting stimulation and sham conditions during NREM sleep. Left and right hand stimulation showed increases in amplitudes in response to stimulation compared to sham conditions that peaked at fronto‐central sites. Top row: Headplots on the indicate the contrasts between stimulation and sham trials, and the contrast between left and right hand stimulation. The color‐coded contrasts represent the ratio of the absolute value of potentials (in microVolts) between left hand stimulation and sham, right hand stimulation and sham, and between left and right hand stimulations. Bottom row: Effect sizes, quantified by Cohen's *d* values indicate comparable increases between left and right hand stimulation compared to the sham condition.

We examined the evoked potentials in response to stimulation (Figure 5). The magnitude of the amplitudes averaged over the 8 s of stimulation significantly differed between stimulation and sham conditions. Combining the left and the right hand trials (*T*_sum_ = 67.9; cluster level *p* value = .002, CI_range_ = 0.002), and contrasting separately the left hand (*T*_sum_ = 42.6; cluster level *p* value = .002, CI_range_ = 0.003), and the right hand (*T*_sum_ = 70.9; cluster level *p* value = .002, CI_range_ = 0.003) trials with the sham condition demonstrated increased amplitude in response to VTS. The cluster included all channel locations except the T6 and O1, and the O1, O2, T4, T6, and the O1, O1, T6 electrodes, in case of the left‐ and right‐sided stimulation (combined), the right‐sided stimulation, and the left‐sided stimulation, respectively. Differences across stimulation and sham trials peaked over fronto‐central sites, and according to the visual representation of ERPs were more pronounced in the first 3–4 s (see Figures 4 and 5). Cohen's *d* effect sizes differentiating left‐ and right‐sided stimulation from sham trials were comparable varying between 0.4 and 0.6 (Figure 6), and no significant clusters emerged between left and right hand stimulation. We compared the differences between the evoked responses to the targeted and untargeted hemispheres. The magnitude of evoked potentials was averaged across electrodes (within the hemispheres) and each value was corrected with the magnitude of evoked responses in the corresponding hemispheres during the sham conditions. We found no significant differences between the targeted and untargeted hemispheres over the 8 s long stimulation period (largest cluster *T*_sum_ = 105.6; cluster level *p* value = .2, CI_range_ = 0.03) providing no evidence against the global increase in amplitudes, regardless of the side of the stimulation.

In the resting wake condition, the magnitude of the amplitudes averaged over the 8 s of stimulation significantly differed between stimulation and sham conditions. Combining the left and the right hand trials (*T*_sum_ = 87.33; cluster level *p* value < .001, CI_range_ = 0.002), and contrasting separately the left hand (*T*_sum_ = 74.44; cluster level *p* value < .001, CI_range_ = 0.002), and the right hand (*T*_sum_ = 42.79; cluster level *p* value < .001, CI_range_ = 0.002) trials with the sham condition clearly indicated an increase in amplitudes in response to VTS. Cohen's *d* effect sizes differentiating left‐ and right‐sided stimulation from sham trials were comparable varying between 0.2 and 0.4. Further examination of the ERPs along the temporal dimension indicated that differences across the stimulation and sham conditions were mainly driven by an amplitude modulation approximately 200 ms after the onset of the stimulation that lasted approximately 500 ms (see Figure S3). In order to compare directly the evoked responses to VTS across sleep and wake, we contrasted the absolute values of the baseline‐corrected amplitude fluctuations of the two conditions. Similar to the above procedure, we averaged evoked responses of left and right hand trials, and compared the magnitude of the amplitudes averaged over the 8‐s long stimulation period by cluster‐based permutation statistics. The magnitude of the evoked response was significantly higher in sleep compared to wakefulness (*T*_sum_ = 50.06; cluster level *p* value = .002, CI_range_ = 0.003, Cohen's *d*_range:_ 0.87–1.43), and the cluster included all electrodes except O2 and T6 locations. Further examination of the temporal dimension of the magnitude of ERPs indicated that VTS triggered higher evoked responses in the sleep than in the wake group, specifically during the first 6 s of stimulation. The contrast during the first 6 s is in line with the pattern observed in sleep showing a sequence of evoked slow waves during the first 6 s. (See Figure 5 indicating that slow waves were entrained in the first 6 s, but were desynchronized after.)

### 3.6 The repetition of vibro‐tactile stimulation did not influence stimulation‐dependent changes in EEG power

The order of the 8‐s long trials did not appear to have a significant influence on the effect of stimulation compared to the sham condition, neither for slow nor for fast frequency EEG power changes (both interaction terms Order × Condition and Order × Band × Condition were not significant). Notably, apart from the effect of Condition (due to larger EEG power changes in stimulation vs sham trials), a significant interaction of Condition × Band appeared, reflecting more pronounced changes in slow power compared to fast frequency power in response to stimulation. Similar to the results of the time‐frequency analyses where we averaged all trials, slow and fast frequency EEG power changes in the mixed‐model analyses where we used slow and fast frequency power changes of each trial separately, we did not observe lateralized effects as a function of the targeted side. The predictors and statistical parameters of the two mixed‐models are summarized in Table 2.

**TABLE 2:** Linear mixed‐effects models predicting slow and fast frequency EEG power changes in each trial

(A)			
Effect	*df*	*F*	*p*
Order	15,223.21	3.569	.059
Condition	15,310.51	23.174	<.001
Band	15,303.33	24.869	<.001
Order × Condition	15,310.93	0.625	.429
Order × Band	15,303.33	0.264	.608
Condition × Band	15,303.33	4.864	.027
Order × Condition × Band	15,303.33	1.236	.266

(B)			
Effect	*df*	*F*	*p*
Order	15,159.09	5.058	.025
Band	15,303.36	35.633	<.001
Site (Targeted vs. Untargeted)	15,305.30	0.156	.693
Order × Band	15,303.36	2.245	.134
Order × Site	15,307.82	1.066	.302
Band × Site	15,303.34	0.006	.937
Order × Band × Site	15,303.36	0.374	.541

*Note for panel A*: All trials (Stimulation and Sham), 5324 observations from *N* = 14 (random grouping factor). AIC: 29,997.927, BIC: 30,063.727.

*Note for panel B*: Stimulation trials only, 5324 observations from *N* = 14 (random grouping factor). AIC: 30,082.345, BIC: 30,148.145.

## 4 DISCUSSION

The aim of our study was to explore the potential of lateralized VTS to boost slow frequency EEG power during NREM sleep, and to examine whether a lateralized stimulation produces relatively higher increases over the targeted compared to the untargeted hemisphere, that is, a local stimulation effect. Our findings indicate that 8‐s long, rhythmic VTS influences cortical activity as measured by EEG. More specifically, we observed an increase in slow frequency power in response to stimulation that peaked between 1–4 Hz, but extended to 13 Hz and showed fronto‐central topographical dominance and a pronounced antero‐posterior gradient. In addition to the boost in slow frequency activity we also observed a widespread increase in fast frequency power between 14–22 Hz that did not show a clear antero‐posterior gradient, peaked over central sites, and was extended to posterior locations. ERP analyses also indicated that our stimulation procedure boosted (~1 Hz) slow waves, that is, increased the amplitude of slow waves in response to the stimulation. These changes in power and amplitude fluctuations were more prominent in the first 3–4 s of repetitive stimulation. On the other hand, the above changes appeared globally, and we did not detect any difference (lateralized effect) between the hemispheres as a function of the stimulated side. A sustained increase in slow frequency power and evoked slow waves in response to VTS also appeared to some extent in resting wakefulness, but was more pronounced in NREM sleep, suggesting that the entrainment of oscillatory activity is more efficient if it is harmonious with the neurophysiological activity of the current state.

Our findings are in line with the growing number of studies showing that slow frequency power can be increased by external stimulation during NREM sleep (Bellesi et al., 2014). Although the acoustic domain is the most trivial modality to enhance slow frequency activity, our results corroborate previous studies indicating that the sleeping brain is also responsive to stimulation in other modalities (Riedner et al., 2011). Such neural responses have a somewhat paradoxical feature: if external stimulation is applied with low intensity, it is able to promote slow wave activity; such slow wave activity appearing in isolation or in transient bursts indicates the instantaneous deepening of sleep, that may indicate the instant promotion of the homeostatic, restorative properties of NREM sleep (Cash et al., 2009; Halász, 2016; Halasz & Bodizs, 2012). Our VTS procedure applied during nocturnal NREM sleep appeared to boost EEG power in a cluster of slow frequencies that peaked within the delta range but extended to higher frequencies. Moreover, the increase in slow frequency power showed an antero‐posterior gradient, indicating a region‐specific response after stimulation. Interestingly, we observed a fluctuation of peaks in antero‐posterior gradients that appeared to follow with a short delay of about 0.5 s the individual pulses of VTS, resembling the nature of traveling slow waves (Massimini et al., 2004). The 1–13 Hz frequency range overlaps with the frequencies that show relative increases in power during recovery nights after sleep deprivation (Marzano et al., 2010), indicating that the homeostatic pressure of sleep does not exclusively impact (1–4 Hz) delta power but also higher frequency components in NREM sleep (Borbély et al., 1981; Marzano et al., 2010). In addition, sleep deprivation affects the EEG spectrum in a region specific manner, as during the recovery night, the increase in slow frequency activity peaks at fronto‐central locations (Marzano et al., 2010) overlapping with the antero‐posterior gradient we observed in case of the cluster of slow frequencies. Large slow waves peaking over fronto‐central sites and coupled with a higher range of EEG components appear spontaneously during NREM periods of high homeostatic pressure, but can also be elicited by external stimulation (Ferri, Bruni, Miano, Plazzi, & Terzano, 2005; Ferri, Bruni, Miano, & Terzano, 2005; Halász et al., 2014). Future studies may examine if elicited slow waves induced by VTS reflect a reactive homeostatic process (Halász et al., 2014).

The inspection of ERPs in response to stimulation showed an increase in slow waves as quantified by the amplitude fluctuations of potentials time‐locked to the onset of stimulations. Interestingly, both the changes in power and amplitudes indicate that the effect of stimulation was more pronounced in the first 3–4 s. This pattern is in line with other studies evidencing a refractory‐like period following the instantaneous increase in slow frequency power (Ngo et al., 2015; Papalambros, Malkani, et al., 2017). For instance, a previous study using 12 s long stimulation sequences that consisted of bursts of pink noise delivered with a rate of 1 Hz, also observed an increase in slow frequency power particularly during the first 4 s of the stimulation trials (Simor et al., 2018). Our control study applying the same VTS during resting wakefulness indicates that slow waves time‐locked to vibro‐tactile pulses and induced slow frequency power are more efficiently boosted during NREM sleep when such oscillations are abundant, in contrast to wakefulness during which slow frequency activity is, albeit present, less dominant (Siclari & Tononi, 2017).

The effect of VTS was not limited to the increase in slow frequency activity, but also enhanced EEG power in the beta (~14–22 Hz) frequency range. The higher frequency cluster exhibited a different topographical distribution compared to the slow cluster as it was less prominent over frontal sites, expressing a less pronounced antero‐posterior gradient. The increase in higher EEG frequency power may indicate transient microarousals that often co‐occur with frontally dominant slow waves and seem to propagate from more posterior, sensorimotor regions (Ferri, Bruni, Miano, & Terzano, 2005; Nobili et al., 2011). The increase in higher frequency activity casts some doubts on the utility of VTS to improve sleep quality, as wake‐like high frequency activity might compromise the restorative properties of NREM sleep and may also lead to sleep fragmentation. High‐frequency EEG power during NREM sleep was linked to increased environmental alertness and hyperarousal, as well as to more intense oneiric activity during NREM sleep (Blaskovich et al., 2020; Riemann et al., 2010; Siclari et al., 2018). Therefore, the concomitant increase in slow and fast frequency activities produced by VTS could fuel future studies focusing on information processing during NREM sleep, such as sensory processing, targeted memory reactivation, or the incorporation of external stimulation into dreams. The increase in fast frequency power was also evident during resting wakefulness and is in line with the observation of a short suppression and a sustained rebound activity of beta power following tactile and proprioceptive stimulation during resting wakefulness (Illman et al., 2020). Although the functional similarity between the elicited fast frequency power in NREM sleep and wakefulness cannot be ascertained, we may speculate that fast frequency activity in response to VTS stimulation reflects sensorimotor processing during NREM sleep, as well as in resting wakefulness (Salenius et al., 1997).

We expected a relatively larger increase in power and amplitude over the hemisphere contralateral to stimulated side, but our findings do not provide evidence for a lateralized effect as a function of the side of the stimulation. Slow waves seem to be expressed in a region‐specific manner as a function of pre‐sleep activity involving specific neural networks (Hung et al., 2013). Furthermore, hemispheric asymmetries in slow waves and other frequencies were observed to some extent in sleeping humans (Bódizs et al., 2017; Tamaki et al., 2016). Whereas cortical slow waves are expressed globally reflecting the synchronized activity of a large number of neural ensembles, a growing body of evidence indicates that the homeostatic regulation of slow waves can also act on more localized levels (Krueger et al., 2019). Our stimulation procedure however, did not seem to differentially modulate the targeted versus the untargeted hemisphere. It cannot be excluded that a lack of lateral effect in our study may stem from low power as well as from low electrode resolution. Therefore, future studies involving larges samples and high‐density EEG may investigate further if lateralized stimulation exerts a localized and, presumably minor effect on the targeted cortical sites. Still, our findings are in line with the results of a similar study that applied lateralized rhythmic auditory stimulation during a daytime nap and observed a global increase in slow waves whenever acoustic stimulation was sent to the left or the right ear (Simor et al., 2018). On the other hand, a more recent study evidenced hemispheric differences in slow waves during unilateral olfactory stimulation. Nonetheless, the odors presented to one of the nostrils during NREM sleep were previously linked with a task requiring participants to memorize words presented on the left or the right visual field. According to the authors, unilateral olfactory stimulation might have selectively reactivated hemisphere‐related memories presented during learning before falling asleep (Bar et al., 2020). Moreover, a control experiment indicated that the lateralized effect in this study was not a result of unilateral stimulation per se, because it did not emerge if the unilaterally presented odor was not previously associated with unihemispheric memory processes (Bar et al., 2020). It is thus feasible that local (lateralized) increases in slow waves can only be induced if they are functionally anchored to learning‐related information processing demands. We may speculate that if external stimulation is not linked to cognitive processing (e.g., memory consolidation) as it does not hold any relevance to the sleeper then such a “meaningless” stimulation may induce a diffuse activity in the reticular ascending system, followed by a global cortical response, instead of activating specific neural pathways.

## AUTHOR CONTRIBUTIONS

**Péter Simor:** Conceptualization; formal analysis; investigation; methodology; visualization; writing – original draft; writing – review and editing. **Tamás Bogdany:** Data curation; investigation; methodology; project administration; writing – review and editing. **Rebeca Sifuentes‐Ortega:** Conceptualization; data curation; investigation; methodology; project administration; writing – review and editing. **Antonin Rovai:** Investigation; methodology; resources; software; writing – review and editing. **Philippe Peigneux:** Conceptualization; funding acquisition; investigation; methodology; resources; supervision; writing – review and editing.

## Supporting information


**FIGURE S1** Effects of vibro‐tactile stimulation (VTS) on EEG power changes during resting wakefulness
**FIGURE S2** Vibro‐tactile stimulation (VTS) differences between sleep and resting wakefulness on EEG power changes
**FIGURE S3** Amplitude fluctuations time‐locked to lateralized vibro‐tactile stimulation during resting wakefulness

## Data Availability

The data that support the findings of this study are available from the corresponding author upon reasonable request.
