# Supplementary material for: Lateralized tactile stimulation during NREM sleep globally increases both slow and fast frequency activities
Source: Psychophysiology. 2022 Sep 25;60(3):e14191. doi: 10.1111/psyp.14191 (PMC10078489; doi:10.1111/psyp.14191)
Supplement: Supplementary file 1 — FIGURE S1 Effects of vibro‐tactile stimulation (VTS) on EEG power changes during resting wakefulness FIGURE S2 Vibro‐tactile stimulation (VTS) differences between sleep and resting wakefulness on EEG power changes FIGURE S3 Amplitude fluctuations time‐locked to lateralized vibro‐tactile stimulation during resting wakefulness [file PSYP-60-0-s001.docx]

**Lateralized tactile stimulation during NREM sleep globally increases both slow and fast frequency activity**

Péter Simor, Tamás Bogdány^1-3^, Rebeca Sifuentes-Ortega^2,6^, Antonin Rovai^4-6^, Philippe Peigneux ^2,6^

**/Supplement/**

*1 Institute of Psychology, ELTE, Eötvös Loránd University, Budapest, Hungary.*

*2 UR2NF, Neuropsychology and Functional Neuroimaging Research Unit at CRCN - Center for Research in Cognition and Neurosciences*

*3 Doctoral School of Psychology, ELTE Eötvös Loránd University, Budapest, Hungary*

*4 Laboratoire de Cartographie fonctionnelle du Cerveau (LCFC), ULB Neuroscience Institute (UNI), CUB-Hôpital Erasme, Université libre de Bruxelles (ULB)*

*5 Department of Functional Neuroimaging, Service of Nuclear Medicine, CUB-Hôpital Erasme, Université libre de Bruxelles (ULB)*

1. *UNI - ULB Neurosciences Institute, Université Libre de Bruxelles (ULB), Brussels, Belgium.*
2. *Comparison of stimulation versus sham conditions including the three dimensions (space, frequency, topography).*

To examine if the stimulation had an effect on EEG power we aggregated the left and right sided trials and contrasted them with the sham trials. Cluster based permutation statistics were performed in all dimensions including space, frequency, and time. The minimum number of channels forming a cluster was set to two. A large positive cluster emerged (T_sum_ = 14675; cluster level p value < 0.001, CI_range_ = 0.002, Cohen’s d_range_ : 0.4 – 2.37) that spanned over all electrodes and time range of analysis, and included frequencies between 1 and 8.5 Hz, and between 14.5 and 24.5 Hz.

1. The lateralized effect of stimulation *including the three dimensions (space, frequency, topography)*

To examine if the side of the stimulation had a specific effect on the targeted versus the non-targeted hemispheres, we have contrasted EEG power changes (between 1 to 30 Hz) across the targeted and untargeted hemispheres, including the data of each lateral electrode in the cluster-based permutation tests. That is, if we stimulated the right finger, the so-called targeted hemisphere corresponded to signals measured at left-sided channels (Fp1, F3, F7, C3, T3, T5, P3, O1), and if we stimulated the left finger, the targeted hemisphere corresponded to signals measured at right-sided channels (Fp2, F4, C4, T4, T6, P4, O2), whereas activity in midline channels were not considered in these analyses. Power values of each electrode were considered to map statistically significant clusters at the topographical, frequency and time dimensions. Cluster based permutation tests were performed separately for left-and right sided stimulations (i.e. contrasting targeted vs. untargeted channels in each frequency and time separately for the left and the right sided stimulation trials). No significant clusters were identified that differentiated the change in power values across the targeted and the untargeted hemispheres (right sided stimulation: largest cluster T_sum_ = 755.85; cluster level p value = 0.21, CI_range_ = 0.03; left sided stimulation: largest cluster T_sum_ = 816.81; cluster level p value = 0.18, CI_range_ = 0.02).

**Figure S1. Effects of vibro-tactile stimulation (VTS) on EEG power changes during resting wakefulness.**


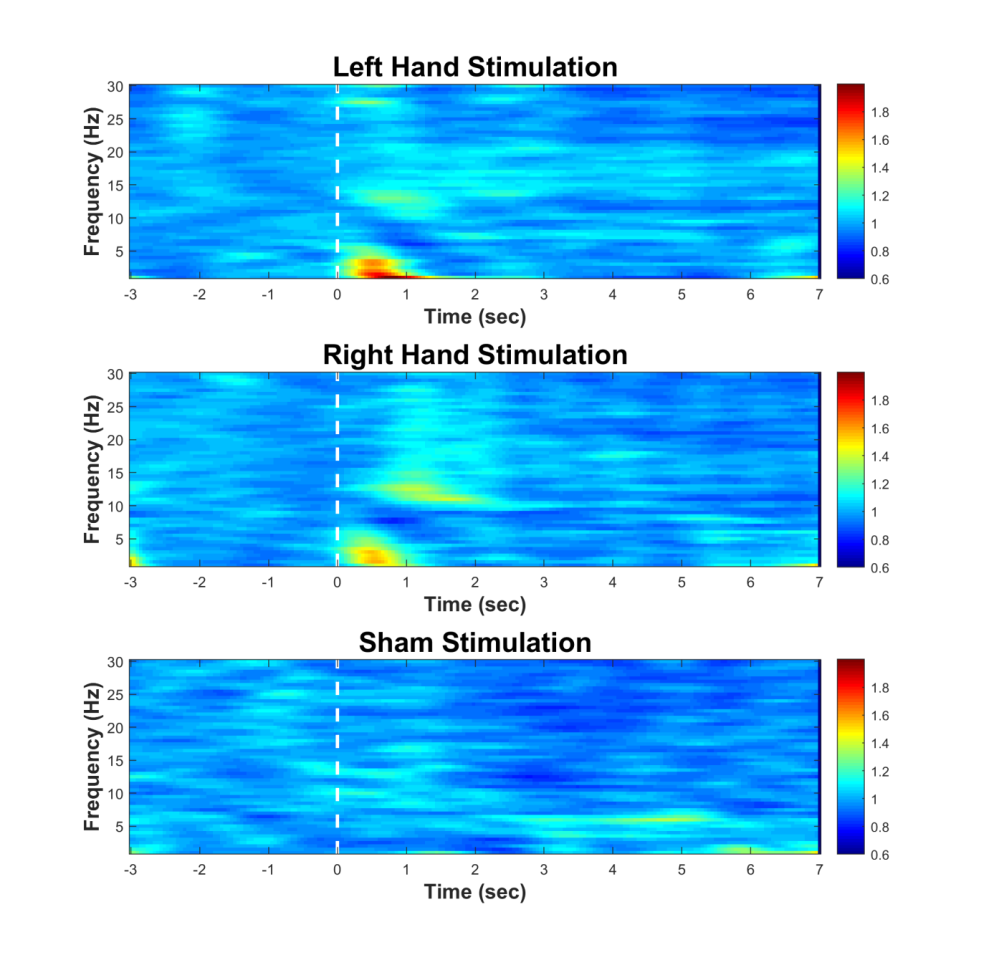


**A)** Time-frequency power changes in wakefulness during stimulation to the left and the right hand, and during sham conditions. The white dashed lines indicate the onset of the trials. An increase in slow and fast frequency power is apparent in both left and right sided stimulation compared to the sham trials. Colour codes between 0.8 and 1.8 represent the power ratio between Stimulation and Sham conditions.


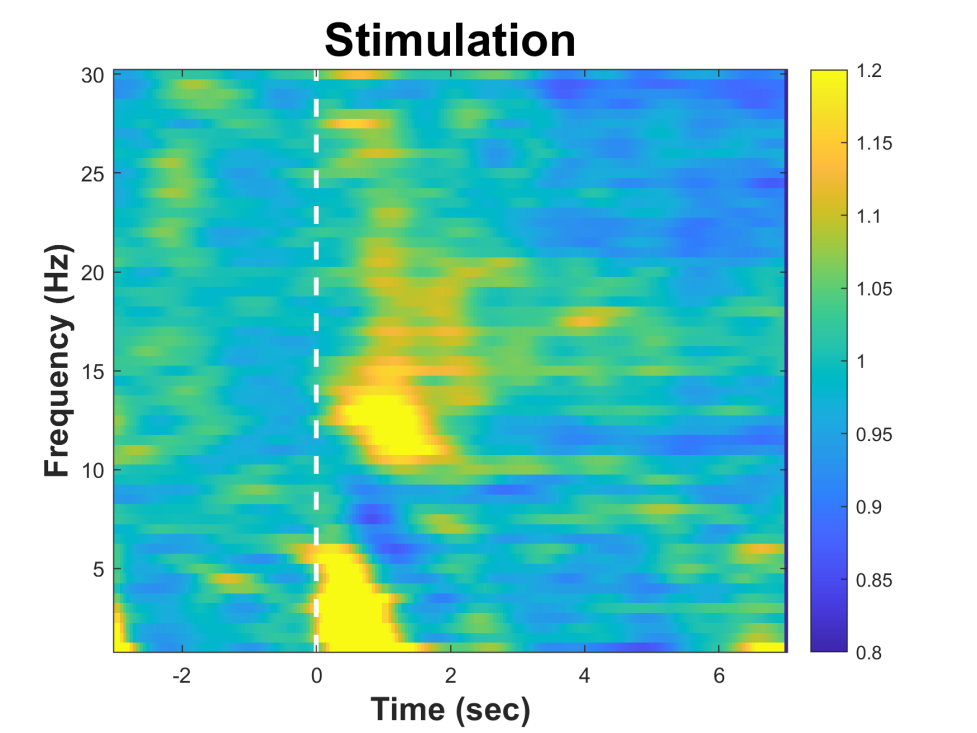


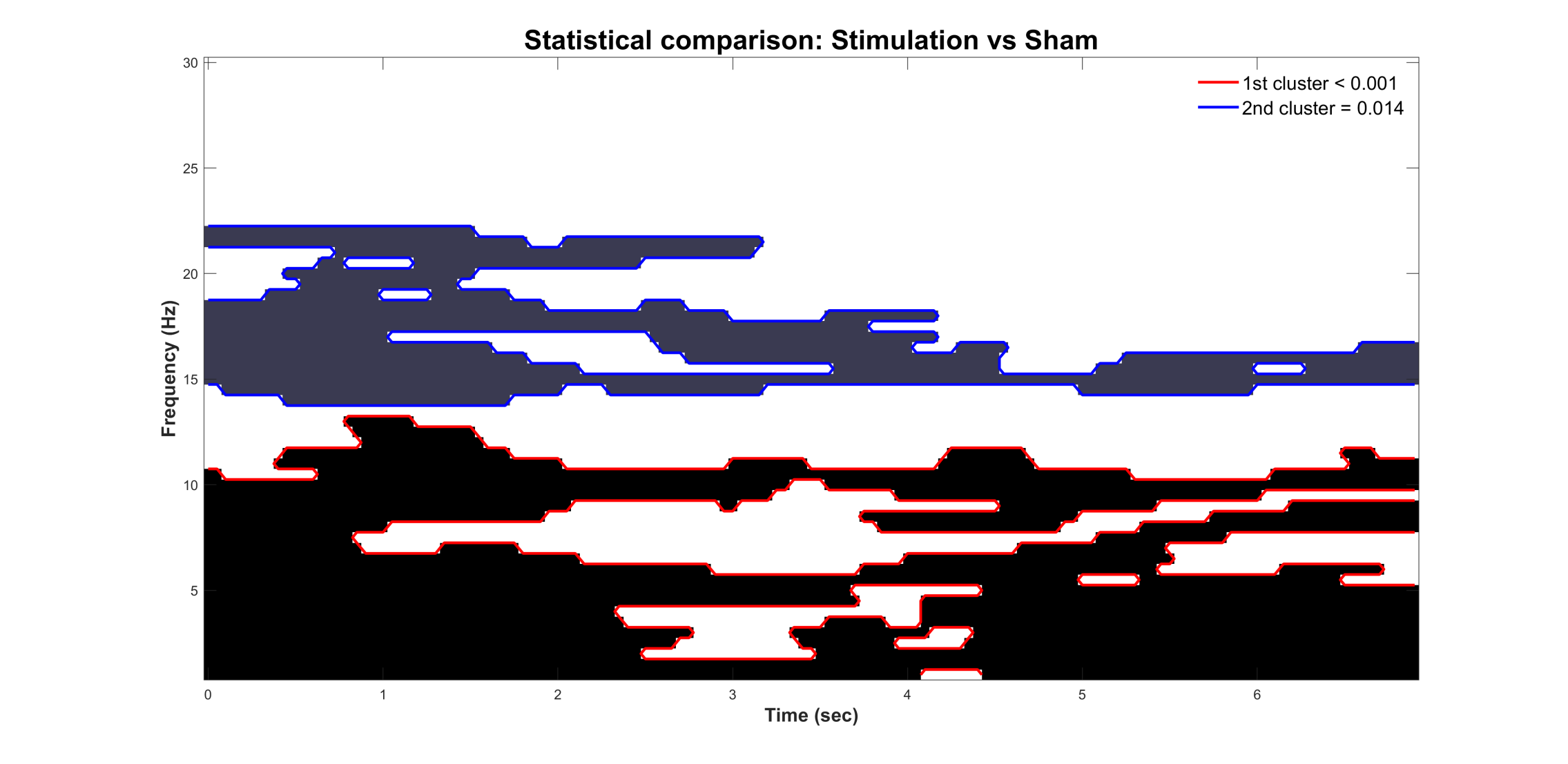
**B)** Time-frequency power changes during stimulation (mean of left and right sided trials) in wakefulness. The white dashed line indicates the onset of the trials.


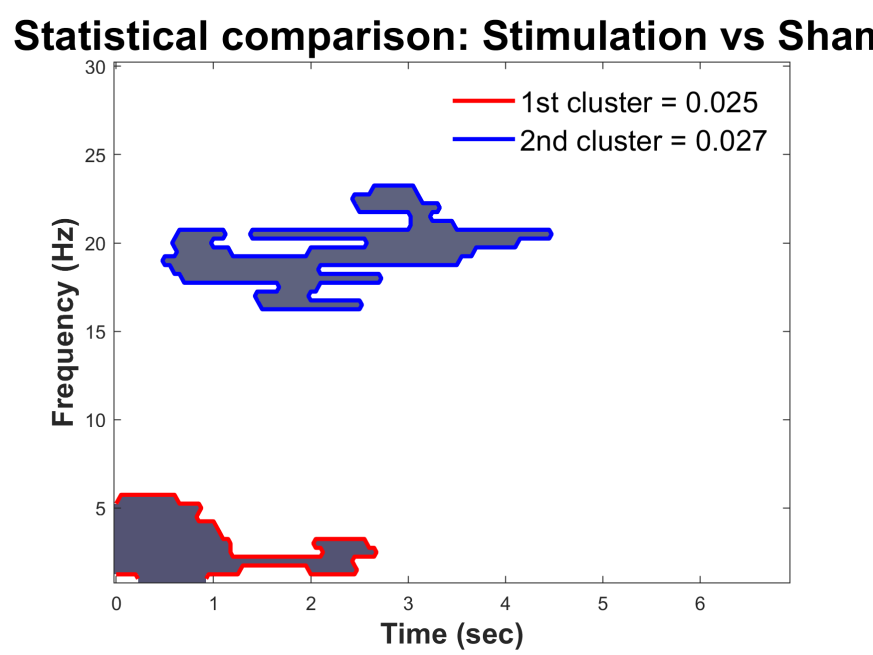


**C)** Clusters in the time and frequency dimensions differentiating stimulation versus sham conditions with respect to EEG power changes after the onset of 8 sec long stimulations in wakefulness.

**Figure S2. Vibro-tactile stimulation (VTS) differences between sleep and resting wakefulness on EEG power changes.**


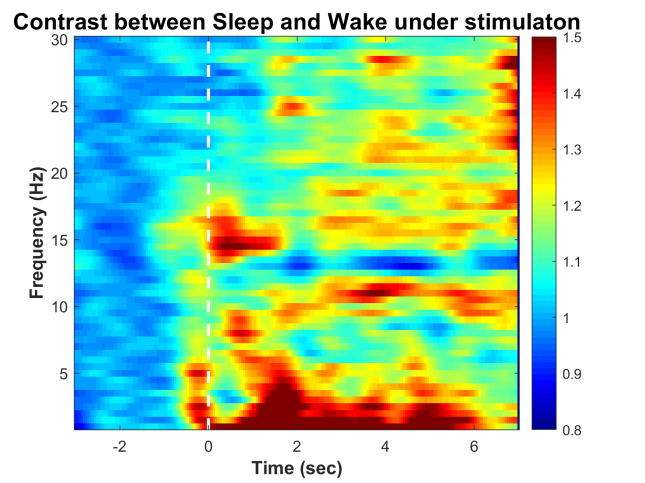


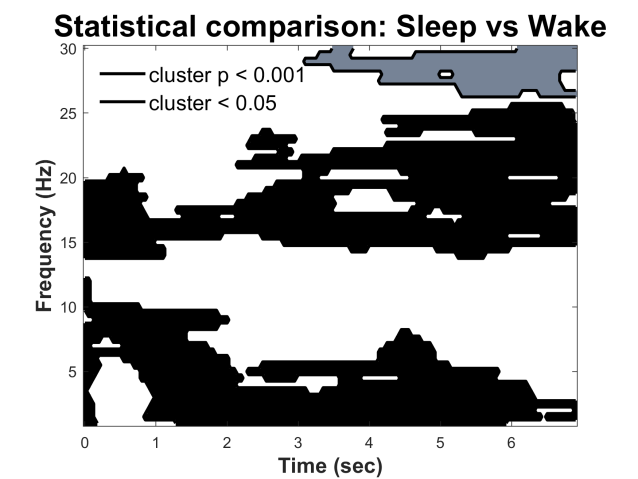


Left panel: Time-frequency power changes during stimulation (to the left and the right hand) in sleep versus resting wakefulness. The white dashed lines indicate the onset of the trials. Colour codes between 0.8 and 1.5 represent the power ratio between sleep and wake groups with regards to the change in power in response to VTS. Right panel: Clusters in the time and frequency dimensions differentiating VTS induced power changes in the sleep versus the wake group after the onset of 8 sec long stimulations.

**Figure S3.** **Amplitude fluctuations time-locked to lateralized vibro-tactile stimulation during resting wakefulness.**


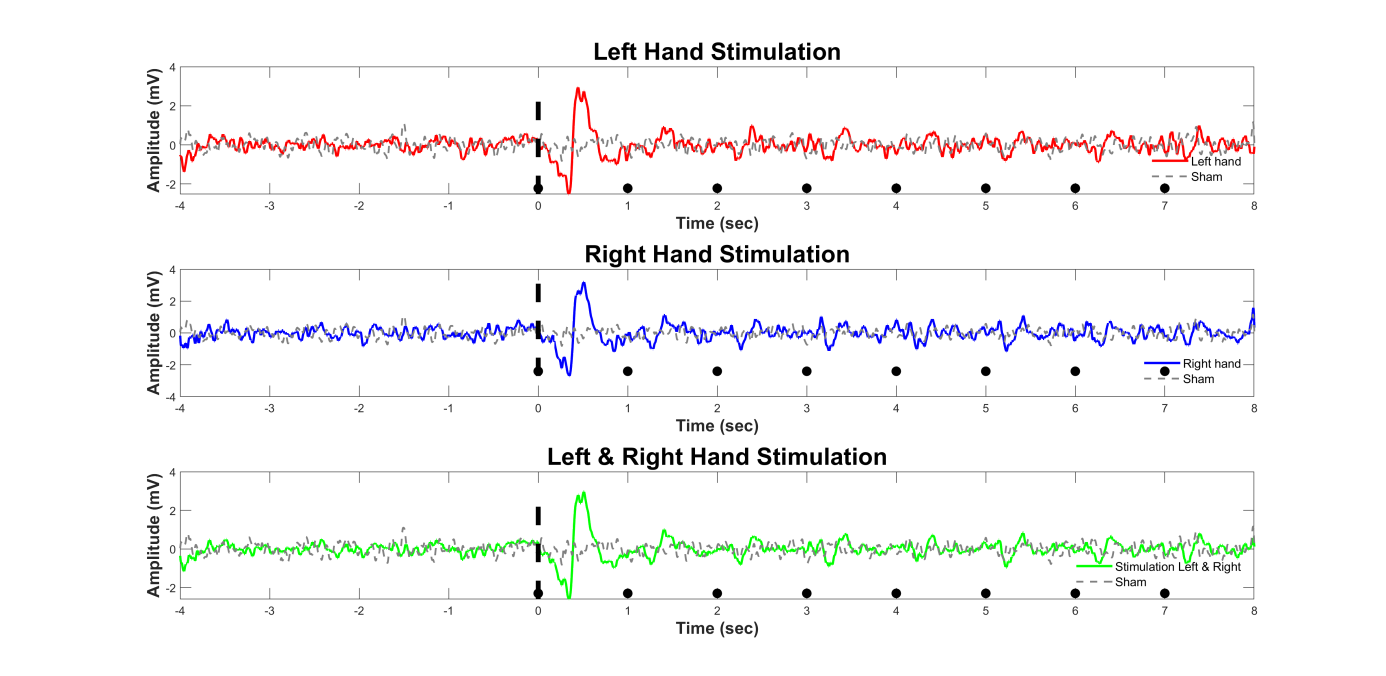


Event related potentials time-locked to left-and right-sided vibro-tactile stimulations contrasted to sham conditions. ERPs are averaged across all electrode derivations. The black dashed vertical lines mark the onset of the stimulation sequences, black points on the x-axis indicate the specific vibro-tactile pulse onsets.
